# Incidental finding of thrombosed aneurysm inside huge pituitary macroadenoma: a case report and review of literature

**Published:** 2012-11

**Authors:** Guive Sharifi, Maryam Jalessi, Kaveh Ebrahimzadeh, Misagh Shafizad

**Affiliations:** ^a^Neurosurgery Department, Loghman Hakim Hospital, Shaheed Beheshti University of Medical Sciences, Tehran, Iran.; ^*b*^Endoscopic Pituitary and Skull Base Surgery Center, ENT-Head & Neck Research Center and Department, Hazrat Rasoul Akram Hospital, Tehran University of Medical Sciences, Tehran, Iran.

## Abstract

Although apoplexy and cystic formation are common inside the pituitary adenoma and hemorrhage with different times of occurrence as revealed by magnetic resonance imaging sequences can be seen within a pituitary macroadenoma, a classic saccular aneurysm and its association with carotid artery haven’t been yet reported.

**Case:**

A 52 years old woman was referred for visual loss and headache accompanied by pituitary adenoma. She has no particular past medical history was operated her huge pituitary macroadenoma was resected by endoscopic endonasal transsphenoidal surgery.

During surgery we encountered a tough saccular mass and checking imaging brought the conjecture of a thrombosed aneurysm. By careful dissection and use of intranasal Doppler sonography its association with left internal carotid was revealed. After coagulation of its main branch that was probably a tumor feeder aneurysm resected totally and we proceed for further tumor removal.

Up to our knowledge and review of the related literatures it is the first time that intra adenoma thrombosed aneurysm is reported.

**Keywords:**

Intra adenoma thrombosed aneurysm, Classic saccular aneurysm, Case report, Doppler Sonography


					Volume 4, Suppl. 1 Nov 2012
Publisher: Kermanshah University of Medical Sciences
URL: http://www.jivresearch.org
Copyright:
Congress of Iranian Neurosurgeons, 14-16 November 2012, Kermanshah, Iran
Paper No. 69
Incidental finding of thrombosed aneurysm inside huge
pituitary macroadenoma: a case report and review of
literature
Guive Sharifi a
, Maryam Jalessi b
, Kaveh Ebrahimzadeh a
, Misagh Shafizad a,*
a
Neurosurgery Department, Loghman Hakim Hospital, Shaheed Beheshti University of Medical Sciences, Tehran, Iran.
b
Endoscopic Pituitary and Skull Base Surgery Center, ENT-Head & Neck Research Center and Department, Hazrat Rasoul
Akram Hospital, Tehran University of Medical Sciences, Tehran, Iran.
Abstract:
Although apoplexy and cystic formation are common inside the pituitary adenoma and hemorrhage with different
times of occurrence as revealed by magnetic resonance imaging sequences can be seen within a pituitary
macroadenoma, a classic saccular aneurysm and its association with carotid artery haven't been yet reported.
Case: A 52 years old woman was referred for visual loss and headache accompanied by pituitary adenoma. She
has no particular past medical history was operated her huge pituitary macroadenoma was resected by endoscopic
endonasal transsphenoidal surgery.
During surgery we encountered a tough saccular mass and checking imaging brought the conjecture of a thrombosed
aneurysm. By careful dissection and use of intranasal Doppler sonography its association with left internal carotid
was revealed. After coagulation of its main branch that was probably a tumor feeder aneurysm resected totally and
we proceed for further tumor removal.
Up to our knowledge and review of the related literatures it is the first time that intra adenoma thrombosed
aneurysm is reported.
Keywords:
Intra adenoma thrombosed aneurysm, Classic saccular aneurysm, Case report, Doppler
Sonography
* Corresponding Author at:
Misagh Shafizad: Neurosurgery Department, Loghman Hakim Hospital, Shaheed Beheshti University of Medical Sciences, Tehran, Iran.
Tel: 09111540432, Email: mishafizad@gmail.com, (Shafizad M.).

				

